# Bullous pemphigoid triggered by COVID‐19 vaccine: Rapid resolution with corticosteroid therapy

**DOI:** 10.1111/dth.15208

**Published:** 2021-11-30

**Authors:** Massimo Dell'Antonia, Speranza Anedda, Federica Usai, Laura Atzori, Caterina Ferreli

**Affiliations:** ^1^ Dermatology Clinic, Department of Medical Sciences and Public Health University of Cagliari Cagliari Italy

Dear Editor,

Bullous pemphigoid (BP) is an acquired autoimmune blistering disease characterized by autoantibodies against basement membrane zone antigens. Several trigger factors have been identified for this disorder, such drugs, ultraviolet radiation, trauma and burns. Pemphigoid cases have been reported in association with vaccination, including rabies,^1^ influenza, pnuemococcus, tetanus, diphtheria,^2^ pertussis, poliomyelitis Heaemophilus influenzae B, hepatitis B and Meningococcus C^3^ vaccine, indicating that vaccination can trigger pemphigoid.

We report here a case of a 83‐year‐old man with a 30 days history of multiple erythemas and blisters with itching. The patient has been affected with hypertension for 20 years, for which he was in treatment with perindopril 10 mg DIE and amlodipine 5 mg DIE. He was otherwise in quite good health with no other significant medical conditions. His family history was negative for significant diseases and skin disorders. The patient developed the first bullous lesions 1 week after the first dose of Comirnaty (Pfizer–BioNTech COVID‐19 vaccine). Blisters were few and located only on his legs and they were initially misdiagnosed as post‐traumatic lesions by his general practitioner. Three days after the second dose of vaccine, which was administrated 1 month after the first, the patient started developing new blisters on his limbs and trunk. When he was eventually admitted to our department he presented several erosions and tense bullae on erythematous base measuring 4–11 cm in diameter on trunk and limbs (Figure 1A). Nikolsky sign was absent and the remainder of the physical examination was normal. Blood exams were carried out, including routine clinical chemistry, neoplastic markers, viral hepatitis markers, autoimmunity panel and quantiferon test. All were within normal limits. Tzank test performed from a fresh bulla showed eosinophils. Histopathology from a bullous lesion demonstrated subepidermal blister with occasional lymphocytes and eosinophilis. Direct immunofluorescence revealed a linear band of C3 along the basement membrane. A diagnosis of bullous pemphigoid was made and the patient treated with topical steroids and prednisone 30 mg daily (0.5 mg/kg daily^4^) for 4 weeks, then reduced by 5 mg every week, then stop. A complete resolution of the blisters was obtained after only 3 weeks of treatment (Figure 1B).

**FIGURE 1:**
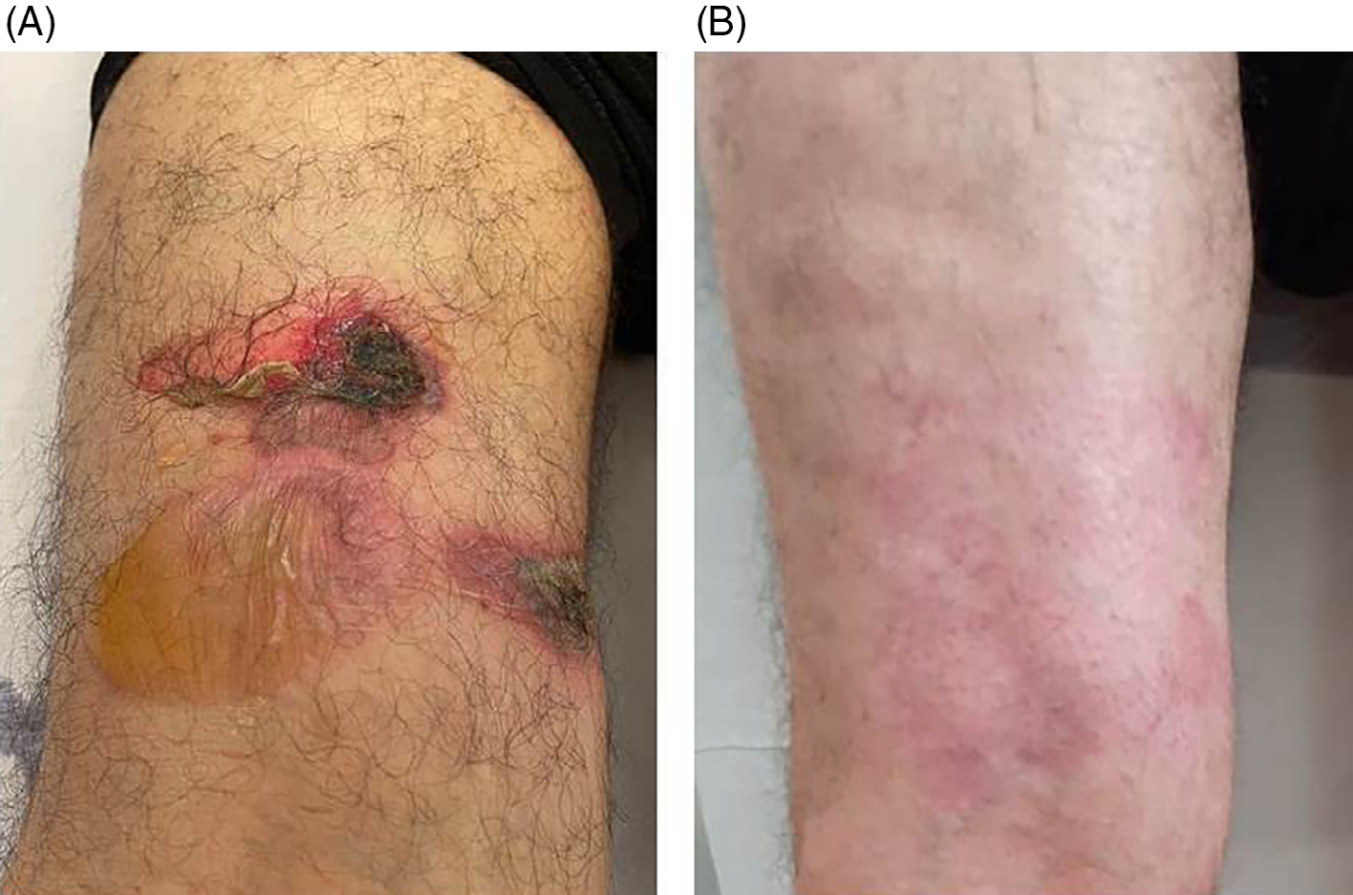
Erosions and blisters on the left leg (A); complete resolution after 3 weeks of treatment (B)

Herein we describe a case of BP triggered by Comirnaty (Pfizer–BioNTech COVID‐19 vaccine). The temporal association between administration of vaccine and appearance of bullous lesion was a pointer that vaccine might be the trigger while reemergence of bullae following the second dose of vaccine confirmed the association. Naranjo algorithm was 9 (definite adverse drug reaction).

Owing to the ongoing COVID‐19 pandemic a mass vaccination campaign has been initiated worldwide. Different types of vaccines are available nowadays.^5^ The adverse reactions occurring with messenger RNA (mRNA) COVID‐19 vaccines mostly appear within the first 30 min after the vaccination and can likely be interpreted as immunoglobulin E‐mediated hypersensitivity.^6^, ^7^ The reported delayed cutaneous adverse events, other than injection site inflammation, are rare. Since a growing percentage of the population becomes vaccinated, delayed cutaneous reactions are likely destinated to increase. To date few cases of delayed cutaneous reactions are reported. Among these, delayed urticaria^7^ and erythema multiforme like eruption, eczematous eruptions, generalized pruritic morbilliform and pytiriasis rosea‐like eruptions, urticarial vasculitis, leukocytoclastic vasculitis^8^, ^9^ and psoriasis exacerbation.^10^ Also few cases of BP are reported.^9^, ^11^

The appearance of blisters after the first administration of Comirnaty should not be not underestimated and carefully considered as an early sign of BP. However, given the risks of SARS‐CoV‐2 infection and the rarity of these events, clinicians should encourage full vaccination.

In our experience, BP triggered by Comirnaty can been easily managed with corticosteroid therapy with a rapid resolution achievable in only 3 weeks.

## AUTHOR CONTRIBUTIONS

All authors contributed equally to the manuscript preparation and revised the final version.

## ETHICS STATEMENT

The patient gave consent for publication of this manuscript.

## Data Availability

The data that support the findings of this study are available from the corresponding author upon reasonable request.

## References

[1] Jindal A , Nayak SUK , Shenoi SD , Rao R , Monappa V . Bullous pemphigoid triggered by rabies vaccine. Indian J Dermatol Venereol Leprol. 2020 Jan‐Feb;86(1):66‐68. doi:10.4103/ijdvl.IJDVL_666_18 31823906

[2] Maki N , Hashimoto T , Yamada T , Ishii N , Tsuruta D , Demitsu T . Case of pemphigoid with immunoglobulin G antibodies to BP180 C‐terminal domain and laminin‐γ1 (p200) developed after pneumococcal vaccination. J Dermatol. 2021;48(1):101‐105. doi:10.1111/1346-8138.15626 32974956

[3] Baroero L , Coppo P , Bertolino L , Maccario S , Savino F . Three case reports of post immunization and post viral bullous pemphigoid: looking for the right trigger. BMC Pediatr. 2017;17(1):60. doi:10.23736/S0392-0488.18.06006-6 28228112 PMC5322655

[4] Cozzani E , Marzano AV , Caproni M , Feliciani C , Calzavara‐Pinton P . Bullous pemphigoid: Italian guidelines adapted from the EDF/EADV guidelines. G Ital Dermatol Venereol. 2018;153(3):305‐315. doi:10.23736/S0392-0488.18.06006-6 29600832

[5] Chung JY , Thone MN , Kwon YJ . COVID‐19 vaccines: the status and perspectives in delivery points of view. Adv Drug Deliv Rev. 2021;170:1‐25. doi:10.1016/j.addr.2020.12.011 33359141 PMC7759095

[6] Shimabukuro T , Nair N . Allergic reactions including anaphylaxis after receipt of the first dose of Pfizer‐BioNTech COVID‐19 vaccine. JAMA. 2021;325(8):780‐781. doi:10.1001/jama.2021.0600 33475702 PMC8892260

[7] Patruno C , Napolitano M , Stingeni L , Fabbrocini G . Skin rashes after SARS‐CoV‐2 vaccine: which relationship, if any? Immun Inflamm Dis. 2021;9(3):622‐623. doi:10.1002/iid3.428 34145775 PMC8342222

[8] Stingeni L , Bianchi L , Zalaudek I , et al. Board members of SIDeMaST. Adverse cutaneous and mucous reactions from anti SARS‐CoV‐2 vaccines: recommendations from the Italian Society of Dermatology (SIDeMaST). Ital J Dermatol Venerol. 2021;156(2):115‐117. doi:10.23736/S2784-8671.21.06992-3 33960747

[9] Larson V , Seidenberg R , Caplan A , Brinster NK , Meehan SA , Kim RH . Clinical and histopathological spectrum of delayed adverse cutaneous reactions following COVID‐19 vaccination. J Cutan Pathol. 2021. doi:10.1111/cup.14104 PMC8444807 34292611

[10] Megna M , Potestio L , Gallo L , et al. Reply to "psoriasis exacerbation after COVID‐19 vaccination: report of 14 cases from a single Centre" by Sotiriou E. J Eur Acad Dermatol Venereol. 2021. doi:10.1111/jdv.17665 PMC8656620 34534379

[11] Tomayko MM , Damsky W , Fathy R , et al. Subepidermal blistering eruptions, including bullous pemphigoid, following COVID‐19 vaccination. J Allergy Clin Immunol. 2021;148(3):750‐751. doi:10.1016/j.jaci.2021.06.026 34275656 PMC8280592

